# Reduced tubuloglomerular feedback activity and absence of its synchronization in a connexin40 knockout rat

**DOI:** 10.3389/fnetp.2023.1208303

**Published:** 2023-08-29

**Authors:** Heather L. More, Branko Braam, William A. Cupples

**Affiliations:** ^1^ Department of Biomedical Physiology and Kinesiology, Faculty of Science Simon Fraser University, Burnaby, BC, Canada; ^2^ Division of Nephrology, Department of Medicine, Edmonton, AB, Canada; ^3^ Department of Physiology, University of Alberta, Edmonton, AB, Canada

**Keywords:** kidney, blood flow dynamics, blood pressure, Wistar-Kyoto rat, Cx40

## Abstract

**Introduction:** Tubuloglomerular feedback (TGF) is the negative feedback component of renal blood flow (RBF) autoregulation. Neighbouring nephrons often exhibit spontaneous TGF oscillation and synchronization mediated by endothelial communication, largely via connexin40 (Cx40).

**Methods:** We had a knockout (KO) rat made that lacks Cx40. One base pair was altered to create a stop codon in exon 1 of Gja5, the gene that encodes Cx40 (the strain is WKY-Gja5^5em1Mcwi^). Blood pressure (BP)-RBF transfer functions probed RBF dynamics and laser speckle imaging interrogated the dynamics of multiple efferent arterioles that reach the surface (star vessels).

**Results:** The distribution of wild type (WT), heterozygote, and KO pups at weaning approximated the Mendelian ratio of 1:2:1; growth did not differ among the three strains. The KO rats were hypertensive. BP-RBF transfer functions showed low gain of the myogenic mechanism and a smaller TGF resonance peak in KO than in WT rats. Laser speckle imaging showed that myogenic mechanism had higher frequency in KO than in WT rats, but similar maximum spectral power. In contrast, the TGF frequency was similar while peak power of its oscillation was much smaller in KO than in WT rats. In WT rats, plots of instantaneous TGF phase revealed BP-independent TGF synchronization among star vessels. The synchronization could be both prolonged and widespread. In KO rats TGF synchronization was not seen, although BP transients could elicit short-lived TGF entrainment.

**Discussion:** Despite the reduced TGF spectral power in KO rats, there was sufficient TGF gain to induce oscillations and therefore enough gain to be effective locally. We conclude that failure to synchronize is dependent, at least in part, on impaired conducted vasomotor responses.

## Introduction

Regulation of renal blood flow (RBF) is dominated by autoregulation, the self-stabilization of perfusion that occurs when blood pressure (BP) varies. In turn, autoregulation is mediated by the myogenic response (MR) that is found in all vascular smooth muscle cells, and the kidney-specific tubuloglomerular feedback (TGF) that operates at the level of individual nephrons. It is TGF that provides the negative feedback that stabilizes RBF and thus renal function when BP varies (Cupples and Braam, 2007). Because the TGF sensor in the distal nephron is the most downstream component of the combined system, the interaction between TGF and the MR is nonlinear.

When acting alone in single nephrons, TGF is an autonomous oscillator (Holstein-Rathlou and Leyssac, 1986). Synchronization of TGF dynamics in nephron pairs that share close arterial connections was first demonstrated in 1987. The uniformly small phase difference between nephrons was consistent only with rapid, vascular signal transmission (Holstein-Rathlou, 1987; Wagner et al., 1997). While the potential explanatory power of synchronization was immediately apparent, its extent and importance could not be assessed until development of laser speckle imagers permitted imaging of perfusion dynamics in multiple star vessels (efferent arterioles that reach the surface). Initial studies using laser speckle imaging confirmed that blood flow, not just tubular pressure, was synchronized in multiple nephrons. While the authors of those studies drew divergent conclusions about the scale of the interaction (Holstein-Rathlou et al., 2011; Brazhe et al., 2014; Mitrou et al., 2015), a recent study has emphasized large temporal and spatial scales of TGF synchronization (Postnov et al., 2022). Collectively, these studies informed two recent reviews (Marsh et al., 2019; Zehra et al., 2021).

Early studies of TGF synchronization established that TGF is a conducted vasomotor response. It acts remotely (Chen et al., 1995; Wagner et al., 1997), enabled by endothelial gap junction transmission of electrical signals (Wagner et al., 1997). We showed that a gap junction blocker, carbenoxolone, impaired TGF synchronization as well as steady state and dynamic autoregulation (Mitrou et al., 2016). This result indicates that TGF synchronization contributes to renal autoregulation. However, gap junction blockers are notoriously pleiotropic drugs and more specific interference with gap junction conductance was needed.

The major endothelial connexin in the kidney, as elsewhere, is connexin40 (Cx40) which provides strong axial conductance to the endothelium (de Wit et al., 2000; Diep et al., 2005; Segal, 2005; Jobs et al., 2012). Absence of Cx40 impairs, but normally does not block, conducted vascular responses in other vascular beds (de Wit et al., 2006; Jobs et al., 2012; Zechariah et al., 2020). Cx40 is also located within the mesangium where it plays significant roles in transmission of TGF signals from the macula densa to the afferent arteriole, affecting vascular resistance, and to the juxtaglomerular apparatus, where it is important to structural organization and tubular regulation of renin secretion (Kurtz et al., 2007; Kurtz et al., 2010). A Cx40 knockout (KO) mouse was generated 25 years ago and has become a mainstay of microvascular research (Simon et al., 1998).

There is little published information concerning TGF in the Cx40 KO mouse. Assessment of TGF by microperfusion in KO mice revealed that the dynamic range of TGF is reduced by 30%–50% from that in wild-type (WT) mice (Oppermann et al., 2013). A study using the isolated juxtamedullary nephron preparation reported that kidneys from KO mice showed no autoregulatory response and essentially no conducted vasomotor response of afferent arterioles (Sorensen et al., 2012), suggesting absence of TGF. No information is available concerning TGF kinetics or dynamics in Cx40 KO mice.

We attempted to perform *in vivo* studies in a Cx40 knockout mouse. It proved impossible to achieve stable BP within the autoregulatory range for long enough to perform relevant studies. Accordingly, we had a Cx40 KO rat made by the Gene Editing Rat Resource Centre at the Medical College of Wisconsin. In this first study we provide initial characterization of the Cx40 KO rat and addressed two questions. Is TGF active and does it contribute to stability of RBF in the Cx40 KO rat? Does TGF in the KO rat exhibit synchronization? Based on the literature we predicted that TGF would active (Just et al., 2009; Oppermann et al., 2013). Based on the literature, impaired synchronization was expected (Jobs et al., 2012; Zechariah et al., 2020), although the extent of impairment was unclear (Sorensen et al., 2012).

## Methods

Experiments were conducted in accordance with the guidelines of the Canadian Council on Animal Care and received prior approval by the Animal Care Committee of Simon Fraser University with continuing evaluation of the new knockout strain. Rats were housed in groups of 2–6 in a 12:12 h light:dark cycle, and had free access to standard rat chow (LabDiet 5,001) and water.

**FIGURE 1:**
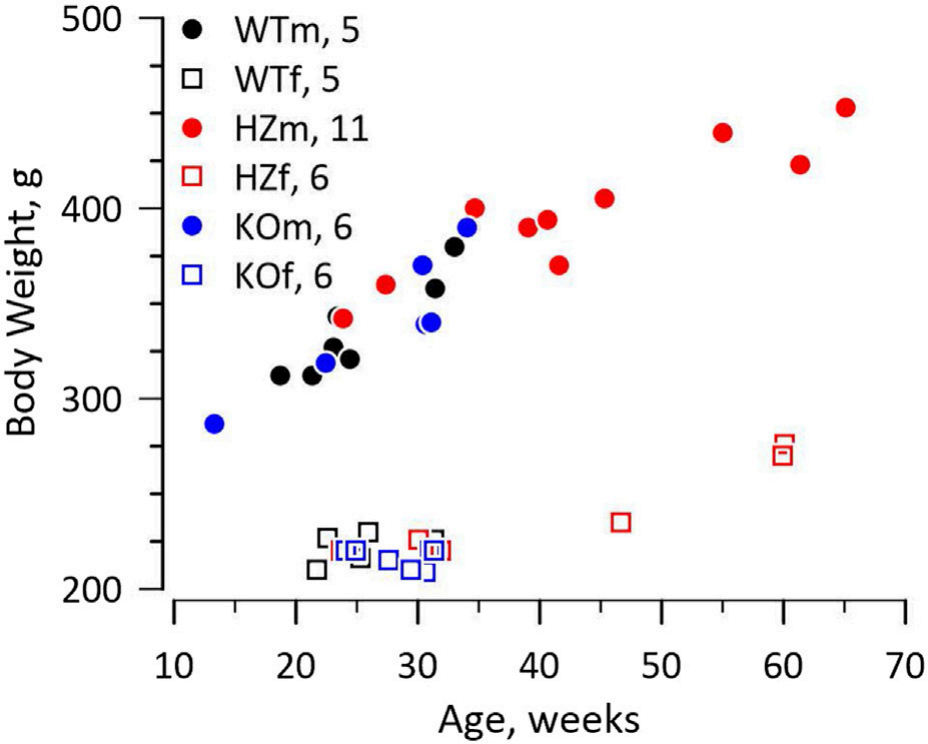
Growth curves. The figure shows the relationship between weight and age for male (m) and female (f) WT, HZ, and KO rats. The numbers of animals for each genotype and sex are reported in the figure. Growth curves differed between sexes as expected, but not among genotypes. The HZ rats tended to be older and therefore larger as some were retired breeders.

The knockout rat was created at the Gene Editing Rat Resource Centre at the Medical College of Wisconsin (https://rgd.mcw.edu/wg/gerrc). A single base change by Crispr introduced a stop codon in exon 1 of Gja5 (WKY-Gja5^5em1Mcwi^). The Wistar Kyoto (WKY) rat was chosen as host because it is a widely used, normotensive, and fecund inbred strain. We received heterozygote (HZ) breeders and continued to breed from HZ parents. Survival of offspring to weaning approximated the Mendelian ratio (21 WT, 50 HZ, 16 KO, χ^2^ = 2.547, *p* > 0.2), including 43 males and 44 females. The WT, HZ, and KO strains had identical growth curves for both males and females, shown in Figure 1. Retired breeders were used for experiments so HZ rats were older and larger than WT and KO rats.

The surgical preparation was described previously (Mitrou et al., 2016). Briefly, rats received buprenorphine (Temgesic^®^ Reckitt & Benckiser, Inc.) 0.01 mg/kg, i.p. 20 min before initiating procedures. Anaesthesia was induced with 4% isoflurane (Baxter, Mississauga, ON, Canada) in inspired air supplemented to 45% O_2_. Anesthetic concentration was reduced to ≈2% during surgery, and 1.25% thereafter. The trachea was cannulated and the animal was ventilated by a small animal respirator (TOPO, Kent Scientific, United States) that operated in timed respiration mode and was adjusted to match the rat’s breathing rate (≈1 Hz). Isoflurane has recently received attention as a vasodilator, particularly in brain, for example, (Lambers et al., 2023). Comparison of data from rats anesthetized by isoflurane, halothane, or Inactin (a barbiturate) reveals no systematic differences in BP, RBF, or conductance in multiple strains, suggesting that vasodilatation by isoflurane is not a major issue in kidney (Cupples et al., 1988; Cupples, 1993; Naguib et al., 1994; Ajikobi et al., 1996; Cupples et al., 1996). In direct comparisons, BP and RBF dynamics are more complex under isoflurane anaesthesia than under halothane anaesthesia (Naguib et al., 1994; Ajikobi et al., 1996; Cupples et al., 1996) and they more closely resemble the dynamics seen in conscious rats (Lessard et al., 1999).

The left femoral vein was cannulated (PE-50) for infusion of 2% charcoal-washed BSA (Sigma-Aldrich, Oakville, ON, Canada) in saline (1% body weight/h). The left femoral artery was cannulated (PE-90 with narrowed tip) and connected to a pressure transducer driven by a Kent Scientific Ltd. TRN005 amplifier. The left kidney was exposed by a subcostal flank incision, liberated from fat and fascia, immobilized in a plastic holder anchored to the table, and covered by a coverslip. After the renal artery was stripped of fat and fascia, a transit-time ultrasound flow probe (PRB-001, Transonic Systems, United States) was mounted and connected to a flowmeter (TS420, Transonic). The flow probe was secured with acoustic coupling gel (NALCO 1181 mixed with surgical lubricant). One hour elapsed between the end of surgery and the start of data acquisition.

Data records had a nominal length of 25 min during which BP and RBF were acquired at 500 Hz, low pass filtered, and resampled to 2 Hz. The relationship between BP and RBF was assessed by transfer and coherence functions. Output of the transfer function consists of gain and phase vectors. The gain vector shows the frequencies at which, and extent to which, BP fluctuation is attenuated in RBF. The phase vector reports the temporal relationship between BP and RBF. A first-order system that responds only to the level of the input variable, BP, has a slope of gain reduction ≈20 dB/decade and a phase peak of π/4 radians. A second-order system responds to both the level of input and its rate of change; it has a slope ≈40 dB/decade and a phase peak of π/2 radians (Milhorn, 1966; Just et al., 1999). The coherence function offers quality control–high coherence at any frequency indicates that the input and output signals are closely and linearly related; reduced coherence can indicate the presence of increased dynamic complexity, noise, or unrelated signals.

Laser speckle images were acquired simultaneously with the BP-RBF records. The acquisition hardware was specified by Dr. DD Postnov, University of Aarhus, who also wrote the drivers and donated them to us. A chosen field of view on the kidney surface was illuminated by a 785 nm, 100 mW laser (Thorlabs, LB875-SF100). Data were acquired by a Basler ACE 2000-165umNIR camera in arrays of 1,000 × 1,000 pixels at 70 Hz. The camera was mounted on an Edmund VZM_300 zoom imaging lens that was in turn mounted on a heavy microscope base that facilitated focusing. This assembly was used at ×2 magnification to give a field of view 2.21 × 2.21 mm (4.88 mm^2^). Blood flow index (BFI) was estimated as the mean light intensity at each pixel over 25 frame intervals divided by its standard deviation, resulting in a 2.84 Hz record (Postnov et al., 2022). The default analyzed segment was full length (1,480 s), but was reduced if necessary.

**FIGURE 2:**
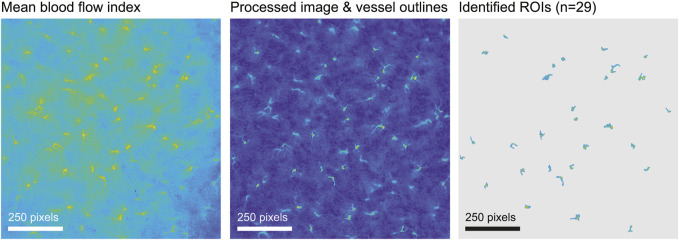
ROI selection. The figure illustrates ROI selection in a WT rat. The left image shows the blood flow index (BFI) image of 1,000 × 1,000 pixels averaged over 2 minutes. In the processed image in the centre, identified ROIs are outlined and the walls of tubules are visible. The image on the right shows the final ROI map.

Regions of interest (ROIs) were extracted automatically from the BFI image using morphological image processing implemented in custom Matlab code, then checked manually. Briefly, we obtained the mean perfusion image, usually over the first 2 min of recording, denoised the image by subtracting the row-wise median and applying a 5-pixel median filter, and adjusted for large-scale differences in lighting with a top-hat transformation. Next we used a modified peak-to-sidelobe ratio to emphasize areas with high perfusion surrounded by areas of low perfusion, and considered pixels with the highest resulting intensity (usually the top 2%) as candidates for star vessels. Morphological opening removed small unconnected areas, after which we kept only areas with the highest intensities (usually the upper 60%) and which were above a size threshold (usually 100 pixels). We added surrounding pixels to the identified areas if they exceeded a threshold intensity (usually 0.5). Finally, we smoothed vessels and filled any holes. Subsequent manual editing of machine-identified ROIs was based on capturing high blood velocity in an efferent arteriole while minimizing capture of much slower blood velocity in neighbouring capillaries and static speckle in proximal tubules (Postnov et al., 2020; Zehra et al., 2021). In WT and HZ rats the TGF peak in ROI spectra is consistent and was used to further differentiate actual from factitious ROIs. In KO rats there was no *a priori* expectation of clear TGF signals so use of ROI spectra was not appropriate. For KO rats we used the same machine-identification of ROIs as in WT and HZ rats with intensity criteria at least as stringent, with subsequent manual selection informed by the experience gained from WT and HZ rats. An example from a WT rat is shown in Figure 2. The median (range) of included ROIs was 27 (18–36) per rat and did not differ by sex or strain. The BFI vector was constructed from the average over all pixels in a ROI at each time and was used for further analysis.

Initial analysis generated figures showing instantaneous BFI of all ROIs and their power spectra. As previously, frequencies of the myogenic mechanism (f_MR_) and TGF (f_TGF_) were machine-extracted from each ROI as the frequency with maximum power within the MR and TGF bands (0.09–0.25 Hz and 0.015–0.05 Hz respectively) (Scully et al., 2013; Mitrou et al., 2015). This analysis found a surprising number of ROIs (307 of 960) with f_TGF_ at the lower limit of the TGF band. The incidence was similar in all groups (30% in WT, 33% in HZ, and 33% in KO rats) and was not sex-related. This suggested a systematic measurement issue unrelated to Cx40. The primary determinant of f_TGF_ is the length of the tubule from the glomerulus to the macula densa in the early distal tubule; a secondary determinant is the transmission time required to send a signal from the macula densa to the afferent arteriole (Holstein-Rathlou and Marsh, 1990). Thus, it is plausible that the KO rat would have lower f_TGF_ than WT and HZ rats, but it is implausible that all strains would have the same high incidence of f_TGF_ <0.02 Hz. We are aware of two potential confounds that could generate this very low frequency signal. First, there is an autonomous oscillator that operates in rat kidneys at ≈0.01 Hz (Siu et al., 2009). Second, this region of the power spectrum often displays 1/f characteristics from which it is difficult or impossible to extract a discrete peak. We were concerned that carte blanche acceptance of the very slow peak power might capture something other than TGF.

Inspection of ROI spectra showed that most ROIs having dominant 0.0176 Hz peaks also had one or more distinct oscillations within the TGF band. We therefore defined criteria for acceptance of f_TGF_: 1) a peak at the lower, or upper, limit of the TGF detection band must be distinct and not part of a 1/f sequence or of a peak outside the band; 2) a distinct peak within the TGF band, defined as twice the noise power, always replaced the peak at or beyond the limit. Noise power is the average power within 0.3–0.5 Hz. If neither of those conditions was met then the ROI was excluded. After applying the criteria, only 28 of 960 ROI had f_TGF_ = 0.0176 Hz while 6 were excluded. The same criteria, with relevant lower and upper limits, were applied to assigning f_MR_, where they had much less impact. For this analysis the MR band was set at 0.09–0.21 Hz to minimize interference by irrelevant BP power >0.2 Hz which is common in these rats. Because distributions of spectral powers in the myogenic and TGF bands appeared to be log-normal, they were log-transformed to normalize variance prior to statistical analysis.

We computed the instantaneous phase of TGF in each ROI using the Hilbert transform applied to the BFI vector. To assess synchronization of TGF we examined plots of instantaneous TGF phase in all ROIs. We considered TGF to be synchronized if plots satisfied 3 criteria: 1) continuing aligned TGF cycles within part or all of the field of view, 2) consistent TGF cycle duration through time, and 3) insensitivity to initiating or driving events in BP. Consistency of TGF cycle duration is included because if ROIs are synchronized then they are phase-locked and will have the same cycle duration, even if they are synchronized in anti-phase.

Samples of kidney cortex were excised, blotted, then immersed in 30% sucrose and OCT for storage at −80°C. They were processed in Braam’s laboratory at the University of Alberta. Frozen 5 µm sections were fixed in acetone for 10 min at −20°C. Slides were blocked in 5% fish gelatin +3.5% BSA +20% donkey serum for 1 hour. They were then exposed to primary antibodies at 4°C overnight. The antibodies used were: anti-Cx40 Invitrogen 364,900 which was used at 1: 100 dilution; anti-smooth muscle actin Novus biologicals NB300-978 which was used at 1:300 dilution; anti-CD31 Thermo-Fisher MA1-81051 which was used at 1:100 dilution. The respective secondary antibodies were Alexa 568, Alexa488, and Alexa647 which were exposed for 1 h. Slides were stored at 4°C in the dark. They were then examined by confocal microscopy.

Data are reported as mean ± standard deviation (SD). Data were first analysed by 2-way analysis of variance (ANOVAn) in Matlab R2022B. Both 3 × 2 (WT, HZ, KO x sex) and 2 × 2 (WT, KO × sex) designs were used to identify sex-related variables and interactions. Because the limited number of replicates was not optimal for these designs, WT × KO (pooled sexes) were also tested by two-tailed t-tests with equal variance. Where there could be ambiguity, the test is reported alongside P. P < 0.05 was considered to indicate a significant difference.

## Results

Table 1 shows that, as expected from the Cx40 KO mouse (Rummery and Hill, 2004; Wagner et al., 2010), BP was higher in the KO rats (*p* = 5.3 × 10^−8^) than in WT and HZ rats which were both normotensive. Although BP appeared lower in the female than the male KO rats, the difference was not significant (*p* = 0.0638). RBF is presented normalized to body weight because there is a tight relationship between body weight and kidney weight in male Wistar rats (left kidney weight (g) = 0.0035 × body weight (g) + 0.148, r^2^ = 0.849 (N = 107), body weight range: 124–519 g, unpublished data). RBF did not differ among strains, although it may have differed between sexes as both HZ and KO females appeared to have lower RBF than WT females (*p* = 0.0703). Renal vascular conductance, normalized to body weight, differed among strains (*p* = 0.0133) and between WT and KO (*p* = 0.0017, t-test).

**TABLE 1:** Basic comparison of WT, HZ, and KO strains.

Strain		N	BW	Age, weeks	BP	RBF	Conductance
WT	All	10	281 ± 65	25.9 ± 4.4	106 ± 10	2.01 ± 0.59	0.019 ± 0.005
	Female	5	222^**a**^ ± 8	25.2 ± 3.7	104 ± 2	2.02 ± 0.51	0.020 ± 0.005
	Male	5	340 ± 28	26.6 ± 5.2	107 ± 15	1.99 ± 0.73	0.019 ± 0.006
HZ^1^	All	17, 16	342^**b**^ ± 82	42.4^**c**^ ± 13.7	102 ± 7	1.85 ± 0.55	0.018 ± 0.005
	Female	6, 5	241^**a**^ ± 25	42.1 ± 15.8	100 ± 7	1.50 ± 0.44	0.015 ± 0.005
	Male	11	397 ± 33	42.6 ± 13.7	103 ± 7	2.01 ± 0.54	0.020 ± 0.005
KO	All	11	274 ± 69	27.7 ± 5.5	127^**d**^ ± 11	1.57 ± 0.49	0.012^**e,f**^ ± 0.003
	Female	7	216^**a**^ ± 5	28.4 ± 3	122 ± 11	1.37 ± 0.28	0.011 ± 0.002
	Male	4	341 ± 36	27 ± 7.7	135 ± 9	1.92 ± 0.61	0.014 ± 0.004

Variables are: **BW**, body weight, g; **BP**, blood Pressure, mmHg; **RBF**, renal blood flow, mL/(min × 100gBW); **Conductance**, (mL/min)/(mmHg×100gBW). Data are presented as mean ± standard deviation.

1For Hz N = 17 for weight, age, and blood pressure, N = 16 for renal blood flow and conductance.

ANOVA, with 3 groups × 2 sexes found differences **a**–**e**. ANOVA, 2 × 2 found difference **f**.

**a**) Body weight differed by sex, *p* = 1.25 × 10^−16^.

**b**) Body weight differed by strain, HZ, being larger, *p* = 0.00036.

**c**) HZ, are older than WT, and KO, *p* = 0.00025.

**d**) Blood pressure differed among groups because it was elevated only in KO, rats, *p* = 5.31 × 10^−8^.

**e**) Weight-normalized conductance differed among the three groups, *p* = 0.0133.

**f**) Weight-normalized conductance differed between WT, and KO, group groups, *p* = 0.004.

**FIGURE 3:**
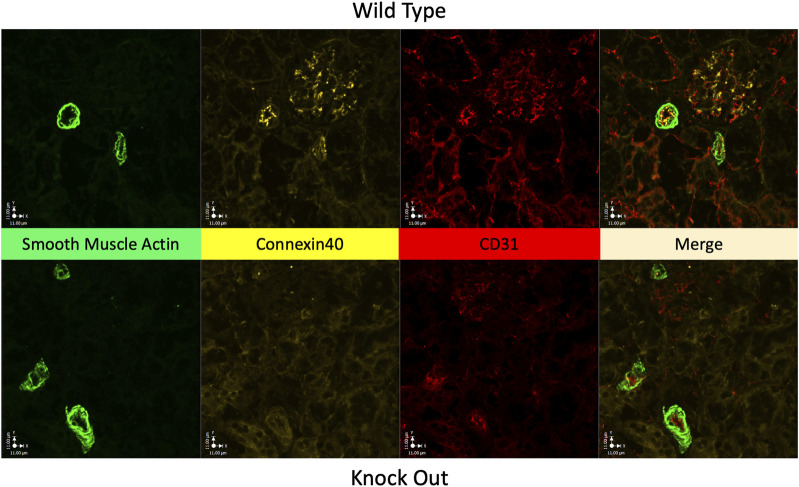
Absence of Cx40 in kidney of KO rat. Immunofluorescence images of renal cortical tissue labelled to show smooth muscle actin (green) a marker of vascular smooth muscle, for Cx40 (gold), and for CD31 (red) a marker of endothelial cells, plus their merged image. Tissue from a WT rat is shown in the upper panels and from a KO rat in the lower panels. In the WT rat, Cx40 is clearly present in endothelium and in the glomerulus, presumably in the mesangium within the glomerulus. Tissue from the KO rat conspicuously lacks Cx40 in both arterial endothelium and within the glomerulus (at 12 o’clock).

Figure 3 shows Immunofluorescence images of renal cortex from a WT rat and from a KO rat. Labelling of Cx40 is clear in the WT sample with punctate labelling of endothelium in 2 muscular vessels and within the glomerulus. In the KO rat 3 muscular vessels and the glomerulus (at 12 o’clock) are evident but, there is no Cx40 labelling.

**FIGURE 4:**
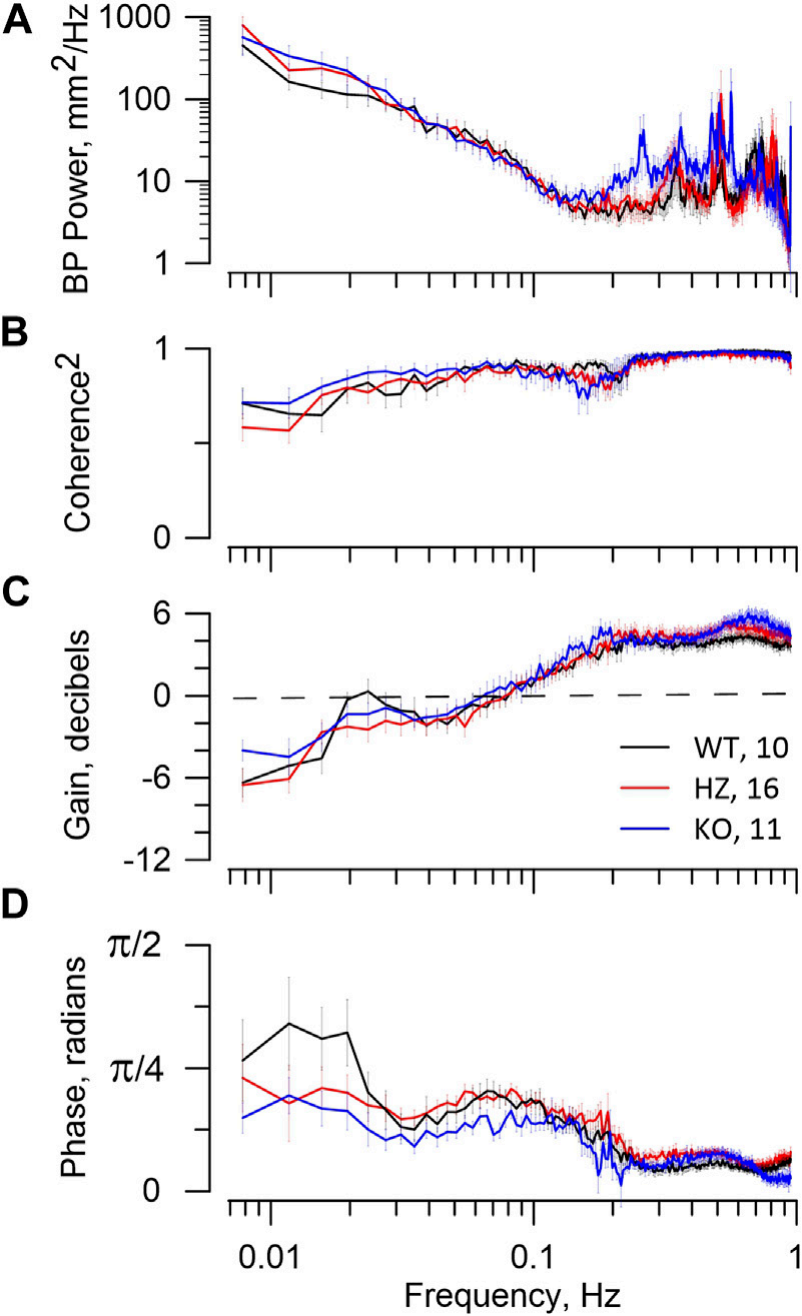
BP–RBF dynamics. The figure reports RBF dynamics in WT, HZ, and KO rats. The figure legend in panel **(C)** includes the numbers of animals used. The top panel **(A)** shows the BP spectral power that is the input to the coherence and transfer functions. The second panel **(B)** shows the expected high coherence at >0.15 Hz, reflecting a tight linear relationship between BP and RBF. At lower frequencies coherence declines slightly. The third panel **(C)** shows the gain vectors. The bottom panel **(D)** shows the phase vectors of the transfer function. The gain reduction that occurs from ≈0.15 to 0.04 Hz and its associated phase peak centred at ≈0.07 Hz are the signature of the MR. Gain <0 dB indicates that RBF is actively attenuating fluctuations from BP. The resonance peak in gain at ≈0.025 Hz and subsequent roll-off at lower frequencies together with the associated phase peaks at <0.02 Hz are the signature of TGF. The MR dynamics indicate weak myogenic autoregulation in all groups. The TGF dynamics indicate adequate autoregulatory capacity in WT, but less so in HZ and KO groups.

Figure 4 presents BP spectral power and RBF dynamics shown in the coherence and transfer functions, with the latter containing gain and phase plots. All strains have similar BP power, i.e., patterns of BP fluctuation. The higher frequency BP power peaks seen in all strains are irrelevant since they do not appear in coherence or gain plots. In all groups the MR operates between 0.15–0.2 Hz with progressive gain reduction to <0 dB at lower frequencies, demonstrating attenuation in RBF of BP fluctuation (autoregulation). All rats display a phase peak centred at 0.07–0.1 Hz that is the temporal signature of the MR. Operation of TGF is shown in WT and HZ rats by the resonance peak in gain centred at ∼0.023 Hz and in the KO rats by a gain shoulder at that frequency. It is also shown temporally in the phase plots with their peak values < 0.02 Hz. There is no reduction of coherence by the MR in any of WT, HZ, or KO rats, indicating that operation of the MR was not sufficient to induce nonlinearity in the BP-RBF relationship. Even the stronger TGF, judged by gain reduction, caused only modest reduction of coherence at frequencies <0.02 Hz.

**FIGURE 5:**
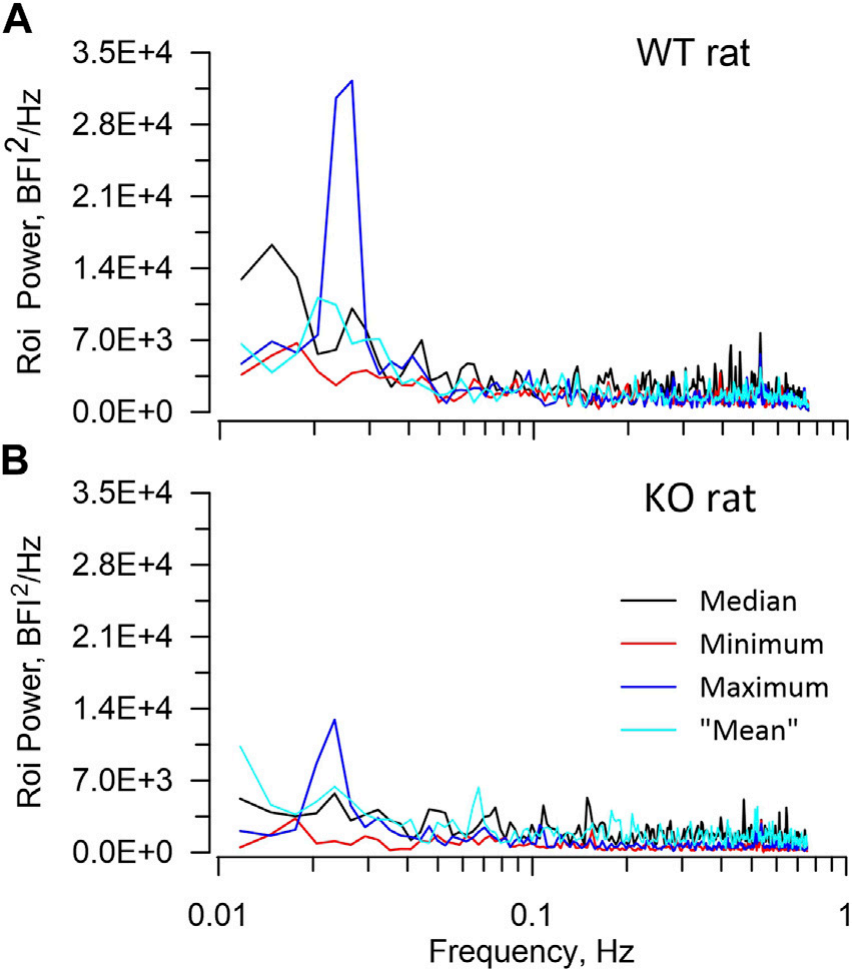
ROI spectra. The figure illustrates ROI spectra extracted from a WT rat **(A)**, upper and a KO rat **(B)**, lower. In each panel spectral powers of 4 ROIs are plotted including the median, the minimum, the maximum, and the “mean”. The “mean” is the ROI that had peak TGF power closest to the true mean. The figure highlights both the lower TGF power in the KO rat and the substantial variability of ROI TGF power that is seen in all rats. Neither rat showed significant power in the frequency band 0.09–0.2 Hz in which the myogenic mechanism operates.

Table 2 presents average ROI areas and numbers by strain and sex. The only significant difference was between male and female HZ rats. As shown in Table 3, TGF power (0.015–0.05 Hz) was greater in WT than in KO rats (*p* = 0.0164). Figure 5 shows median, minimum, maximum, and “mean” ROI power spectra taken from a WT rat and from a KO rat (The “mean” is the ROI that had peak TGF power closest to the true mean for that rat). The figure illustrates the large variability of TGF power seen in all rats and the lack of discrete MR power. It is worth noting that spectral power in the MR band (0.09–0.21 Hz) was minimal in both rats with little evidence of a peak or peaks. We commonly observed this lack of MR power in all groups and the lack of power is reflected in the MR dynamics seen in transfer functions (Figure 4). Table 3 reports peak powers and frequencies of both MR and TGF and average noise power. Peak MR power in the band 0.09–0.21 Hz was only about three-fold higher than the noise power, shown in Table 3. Nor did peak MR power differ between groups or sexes. Instead, f_MR_ differed between WT and KO rats (*p* = 0.0297) with the KO rats having a higher frequency (Table 3).

**TABLE 2:** ROI characteristics.

Strain		ROI area, pixels	Number of ROI/rat
WT	All, 9	436 ± 91	27 ± 5
	Female, 4	438 ± 138	25 ± 6
	Male, 5	435 ± 48	29 ± 3
HZ	All, 16	452 ± 106	26 ± 5
	Female, 6	365* ± 96	23 ± 4
	Male, 10	504 ± 133	28 ± 4
KO	All, 12	421 ± 108	25 ± 4
	Female, 7	401 ± 76	25 ± 3
	Male, 5	448 ± 147	25 ± 4

Data are presented as mean of rats ±standard deviation.

ANOVA, with 3 groups × 2 sexes found a sex difference in ROI, area only between male and female HZ, rats (*, *p* = 0.0446, confirmed by t-test). ANOVA, with 2 groups × 2 sexes did not find any significant differences between WT, and KO, rats or between sexes. Results from HZ, rats are included for completeness and reference. They are not discussed in the text.

**TABLE 3:** MR and TGF variables.

Strain, N	Log(MP_MR_)	f_MR_, Hz	Log(MP_TGF_)	f_TGF_, Hz	Log(noise power)
WT, 9	3.43 ± 0.19	0.132 ± 0.012	4.14 ± 0.45	0.0254 ± 0.0028	3.07 ± 0.20
HZ, 16	3.48 ± 0.21	0.137 ± 0.014	3.99 ± 0.48	0.0277 ± 0.0034	3.05 ± 0.22
KO, 12	3.52 ± 0.23	0.146 ± 0.014*	3.73 ± 0.26**	0.0279 ± 0.0027	3.07 ± 0.24

Data are presented as mean ± standard deviation.

Maximum powers (MP) for MR, and TGF, were acquired in the bands 0.09–0.21 Hz and 0.015–0.05 Hz respectively. f_MR_, and f_TGF_, are the dominant frequencies in those bands at maximum power. Noise power is the average power in the band 0.3–0.5 Hz and did not differ among groups or between WT, and KO, rats. ANOVA (2 × 2) found that Log(MP_MR_) did not differ between WT, and KO, or among groups; f_MR_, was higher in KO, than in WT, rats (*, *p* = 0.0496). Log(MP_TGF_) was lower in KO, than in WT, rats (**, *p* = 0.0235). Results from HZ, rats are included for completeness and reference. They are not discussed in the text.

**FIGURE 6:**
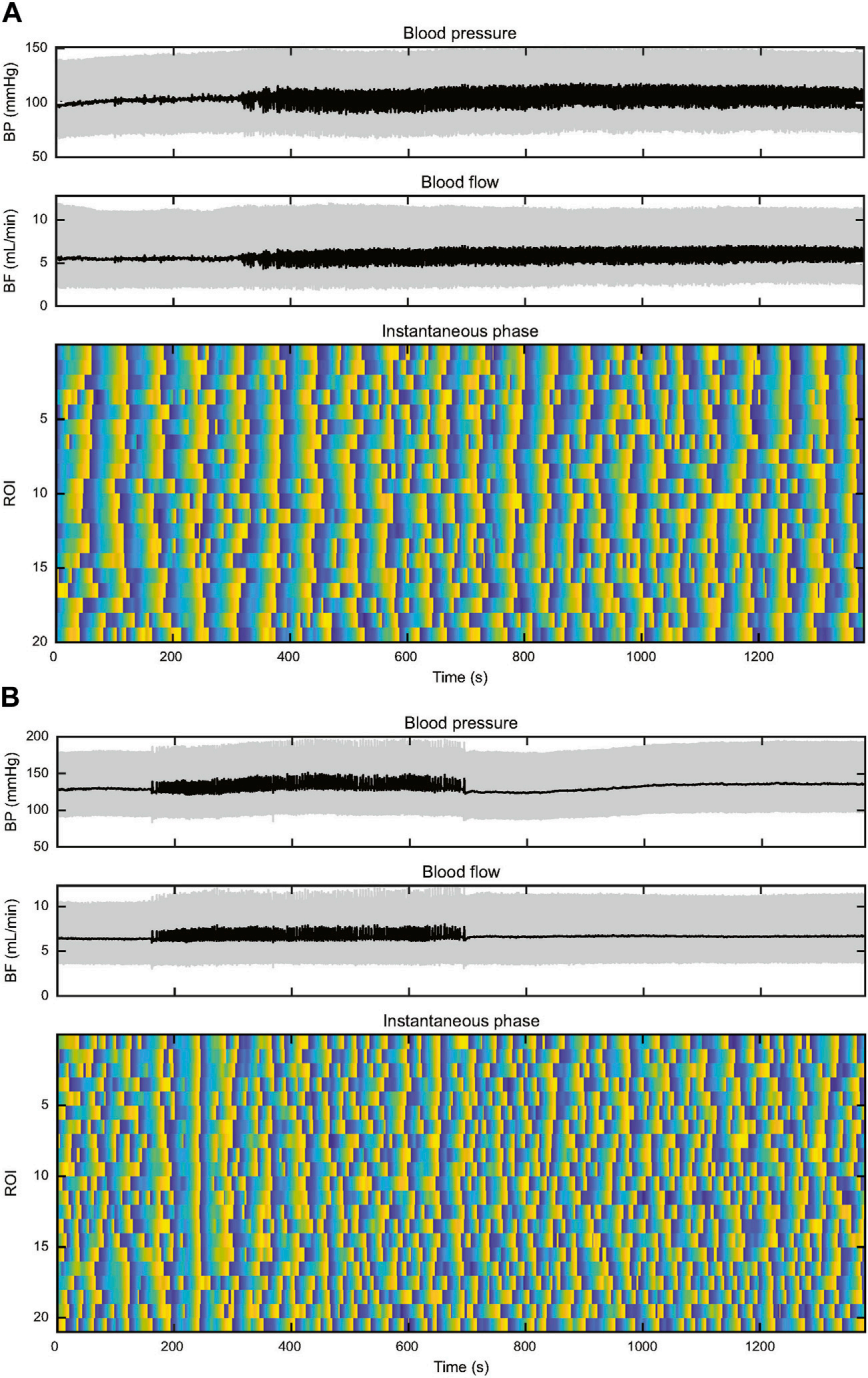
This figure depicts records of BP, RBF, and instantaneous TGF phase from two rats. The upper set of panels **(A)** are from a WT rat and the lower set of panels **(B)** are from a KO rat. In both records, BP is stable throughout the record, both showing episodes of 0.25–0.5 Hz input from the autonomic nervous system. **(A)** The WT rat shows widespread entrainment of instantaneous phase in almost all the 20 ROIs from the start of the record to ≈500 s. Thereafter TGF signature becomes disordered until ≈700 s when synchronization begins again, particularly in ROIs 1–9. The synchronization spreads throughout the field of view before the initiating ROIs become disorganized shortly after 900 s. By 1,100 s the field of view is again fully synchronized. **(B)** In the KO rat there are no BP transients to drive TGF entrainment and the lack of synchronization throughout the record is evident. There is one TGF cycle of full entrainment at ≈230 s, but the entrainment decays within <2 TGF cycles. The rest of the instantaneous phase record shows little evidence of entrainment and a high incidence of variable TGF cycle duration.

Two plots of instantaneous TGF phase are shown in Figure 6, one from a KO rat and the other from a WT rat. Three further examples are shown in Supplementary Material. They illustrate several aspects that separate TGF entrainment from TGF synchronization. TGF entrainment is seen in both WT and KO rats and is a response to BP transients, particularly BP reductions. These alignments were brief, typically decaying over one to two TGF cycles, so they did not differentiate between WT and KO rats. TGF synchronization was observed in 6 of 9 WT rats. The plots of instantaneous TGF phase show episodes of TGF synchronization ranging in duration from 3 TGF cycles to the full record length and in size from a few ROIs to the entire field of view. Here the alignment of TGF phase among ROIs is clearly independent of BP and often robust to BP transients (Figure 6, Supplementary Data). Episodes of synchronization started, grew or shrank, and stopped independently from any observable change in BP or RBF. Of the remaining 3 WT rats, BP-induced TGF activation accounted for all entrainment in two of them and the third displayed 21 transient BP reductions in an 800 s record (≈0.026 Hz) making it impossible to determine the source of TGF entrainment. Synchronization was not observed in any KO rat. Instead, half of KO rats showed short-lived TGF entrainment, typically one to two cycles, which was temporally associated with transient events in BP. The difference in observed synchronization between 6 of 9 in WT and 0 of 12 in KO was significant (χ^2^ = 13, *p* < 0.005).

## Discussion

The Cx40 KO rat is a new model that was created to facilitate studies of TGF synchronization and is relevant to studies of conducted vasomotor responses throughout the body. It offers advantages for integrative studies that address function and its regulation on scales that are larger than are possible in the mouse. The dimensions of synchronized regions reported here and in (Postnov et al., 2022) appear to approach the renal surface area of a mouse kidney.

Most of what we know or surmise about the actions of Cx40 in the kidney was acquired in the Cx40 knockout mouse. Hypertension in the Cx40 KO rat was expected since Cx40 has been shown in the mouse to be necessary for both organization and function of the juxtaglomerular apparatus (Gerl et al., 2015). Other studies of mice have shown that the Cx40 knockout is also a Cx37 knockdown, Cx37 being the other major endothelial connexin (Kurtz et al., 2009; Jobs et al., 2012). It is unlikely that the dominant vascular smooth muscle connexin, Cx45, could substitute for knockout of the endothelial Cx40. This was shown in two studies that used a mouse with the Cx40 coding region replaced by Cx45 to show that Cx45 could only partly restore regulation of renin secretion, without restoring structure of the juxtaglomerular apparatus (Schweda et al., 2009). In addition, it only partly restored autoregulation (Just et al., 2009). The HZ rat is normotensive, indicating that there is adequate communication from the macula densa to the juxtaglomerular apparatus. While we bred from HZ parents, others keep separate WT and KO colonies (DG Welsh, personal communication).

This leads to an interesting consideration for interpretation of Cx40 knockout studies in any species. ANG II augments TGF (Ploth and Roy, 1982; Braam and Koomans, 1995), MR (Cupples, 1993; Kirton and Loutzenhiser, 1998), and autoregulation in conscious rats (Polichnowski et al., 2015), acting at multiple sites within the negative feedback loops. Thus, any effect of elevated [ANG II] in KO rats will tend to minimize differences between WT and KO rats in the context of RBF regulation.

Because BP differed between WT and KO while RBF did not, renal vascular conductance is lower in the hypertensive KO than in WT or HZ rats. There is an apparent sex difference in conductance between WT on one hand and HZ and KO on the other that becomes nonsignificant when conductance is normalized to body weight. Reduced conductance in KO males is consistent with increased BP and autoregulation. This is not the case for KO females or for HZ females in which BP is not the issue. The female to male ratios of body weight are similar, being 0.65 in WT, 0.63 in HZ, and 0.63 in KO rats whereas the female to male ratios of weight-normalized RBF are 1.06 in WT, 0.75 in HZ, and 0.71 in KO rats. Statistical testing may have failed to detect lower RBF in female HZ and KO rats due to insufficient numbers of replicates. Assuming that the relationship between body weight and kidney weight in female rats is at least similar to that in males then we are missing something.

The most important point shown by the BP-RBF transfer functions is that KO rats display reasonably effective dynamic autoregulation, albeit with smaller contribution from TGF than in WT rats. The second point is that coherence declines only slightly at frequencies < f_TGF_, indicating that the system is remarkably linear in these rats. This is intriguing because the TGF sensor at the macula densa is the most downstream component of the combined autoregulation system while both TGF and MR operate on the same vascular smooth muscle. Thus, nonlinearity is to be expected when both systems are operating even if the MR were not being modulated by TGF. Such modulation has been demonstrated repeatedly by investigators using a variety of experimental designs (Chon et al., 2005; Sosnovtseva et al., 2005; Shi et al., 2006; Sosnovtseva et al., 2007; Siu et al., 2009).

One point that we find perplexing is that all these WKY rats display slower TGF-mediated autoregulation than we have reported previously. Over 25 years we used both outbred (Sprague-Dawley, Wistar, Long-Evans) and inbred strains (SHR, Wistar Kyoto, Brown Norway). Almost without exception they exhibited TGF-mediated gain roll-off at ≈0.03 Hz as opposed to the ≈0.02 Hz roll-off seen in the current study. We do not have a satisfactory explanation for this difference, but we feel it is noteworthy.

Dynamics of MR and TGF in ROIs differed between WT and KO rats. The MR in KO rats had a higher f_MR_ but similar maximum power as in WT rats although it was often difficult to separate MR from noise. Interestingly, we were able to identify by eye the MR peaks in ROI spectra from 7 of 12 KO, but only 1 of 9 WT rats, suggesting that the MR was more organized in the KO rats. That plus the higher f_MR_ associated with reduced TGF power suggests release of MR from TGF modulation in KO rats as has been suggested to explain the increased f_MR_ in isolated, perfused hydronephrotic kidneys that have only MR (Marsh et al., 2019). Another possibility would be BP-dependency of f_MR_ (Cupples and Loutzenhiser, 1998).

The lower peak TGF power that is seen in KO rats indicates reduced TGF gain although, to a considerable extent, the oscillations are still present as illustrated in Figure 5. The bulk of TGF studies in rats were performed using barbiturate anaesthetics. Those studies routinely demonstrated TGF gain sufficient to regulate pre-glomerular resistance and contribute to autoregulation (Schnermann and Briggs, 1989; Casellas and Moore, 1990; Braam et al., 1993), even in Cx40 KO mice (Oppermann et al., 2013). In addition, Kallskog and Marsh demonstrated additive TGF interactions in nephron pairs of rats anesthetized by Inactin (Kallskog and Marsh, 1990). Barbiturates, and particularly Inactin, reduce TGF gain to below the critical level that causes bifurcation to oscillating dynamics (Pitman et al., 2004). In turn, this suggests that the residual gain in KO rats in the current experiment is above that critical level, at least in some nephrons, and sufficient to initiate conducted vasomotor responses if all components of such responses are present and active.

We used examination of instantaneous phase plots to identify synchronization according to the criteria stated in Methods. Gap junction mediated communication is certainly not required for BP-dependent entrainment of TGF which was seen in both WT and KO rats. It is, however, the only known communication pathway that enables TGF synchronization. Synchronization was not observed in any KO rat. Instead, TGF entrainment was observed in temporal association with BP transients, usually BP reductions, and was always short-lived, decaying within 1 or 2 TGF cycles. Parenthetically, the presence of widespread BP-induced TGF entrainment is strong evidence that TGF is active or can be induced in most or all nephrons. In contrast to the absence of synchronization in KO rats, 6 of 9 WT rats showed episodic or sustained TGF synchronization that involved either a portion of the field of view or the entire field of view. Both initiation and termination of widespread synchronization were observed in the absence of any BP or RBF perturbation. The duration of synchronization was often in the range of 5–7 min and in one case for 25 min (≥35 TGF cycles), results that are consistent with those of (Postnov et al., 2022). Most of the synchronized ROIs oscillated in phase while a few were clearly synchronized but out of phase.

## Summary and conclusions

We had a Cx40 knockout rat made in which a single base change created a stop codon in exon 1 of Gja5, the gene that encodes Cx40. The KO rats breed and grow normally. They have relatively normal RBF dynamics although the power in TGF oscillations is much lower than in WT rats. Since TGF oscillations are present in KO rats, TGF is undoubtedly operating in individual nephrons. Synchronization of TGF dynamics is seen in most of the WT rats, but synchronization could not be differentiated from BP-dependent entrainment in any of the KO rats. We conclude that the Cx40 KO rat has TGF that is active in individual nephrons, indicating that Cx40 is not essential for transmission of the TGF signal across the extraglomerular mesangium to the afferent arteriole. Cx40 does appear to be essential for vascular transmission beyond the afferent arteriole which would otherwise enable conducted vascular responses and synchronization. Overall, these results argue for large radii of TGF synchronization. The absence of TGF synchronization in Cx40 KO rats is consistent with a reduced radius of interaction due to reduced transmission along afferent arterioles and small arteries.

## Data Availability

The original contributions presented in the study are included in the article/Supplementary Material, further inquiries can be directed to the corresponding author.
