# Supplementary material for: Reduced tubuloglomerular feedback activity and absence of its synchronization in a connexin40 knockout rat
Source: Front Netw Physiol. 2023 Aug 29;3:1208303. doi: 10.3389/fnetp.2023.1208303 (PMC10495682; doi:10.3389/fnetp.2023.1208303)

Supplemental Data

This file contains three pairs of records similar to those shown in figure 6. In each, the sets of panels labelled (**A**) show results of BP, RBF, and instantaneous TGF phase from a WT rat while the sets of panels labelled (**B**) present the same results from a KO rat. Pairs of WT and KO rats were chosen for presentation largely on the basis of similarity in BP records.

SUPPLEMENTAL **Figure 1.A** In this WT rat there is moderate TGF synchronization from the beginning of the record to ≈630 s. During that time the synchronization starts in ROIs 5 – 19 and spreads over to cover the field of view. There are definable BP transients at 200 s, 560 s, 720 s, and 1280 s. Of these, only the event at 730 s induced TGF entrainment. Thereafter TGF synchronization continues in ROIs 1 – 7 and 17 – 27, eventually again spanning all 27 ROIs.


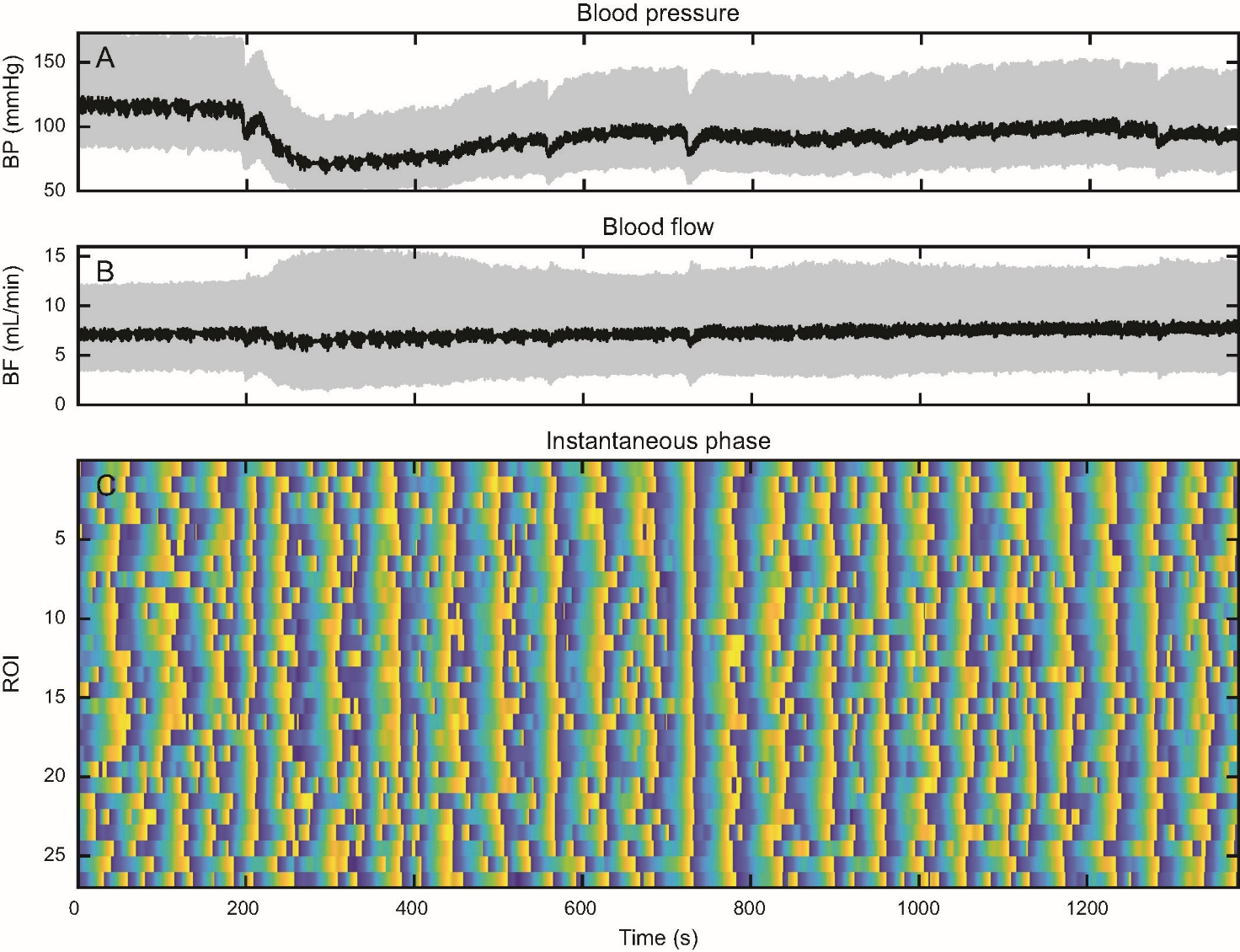


SUPPLEMENTAL **Figure 1.B**) In this KO rat, three small transient BP drops induce entrainment of TGF at 375, 603, and 1120 s. In all cases the entrainment lasts at most 2 cycles. The rest of the record shows little evidence of TGF entrainment. This figure illustrates the sensitivity of TGF entrainment to BP transients in KO rats and the short duration of that entrainment.


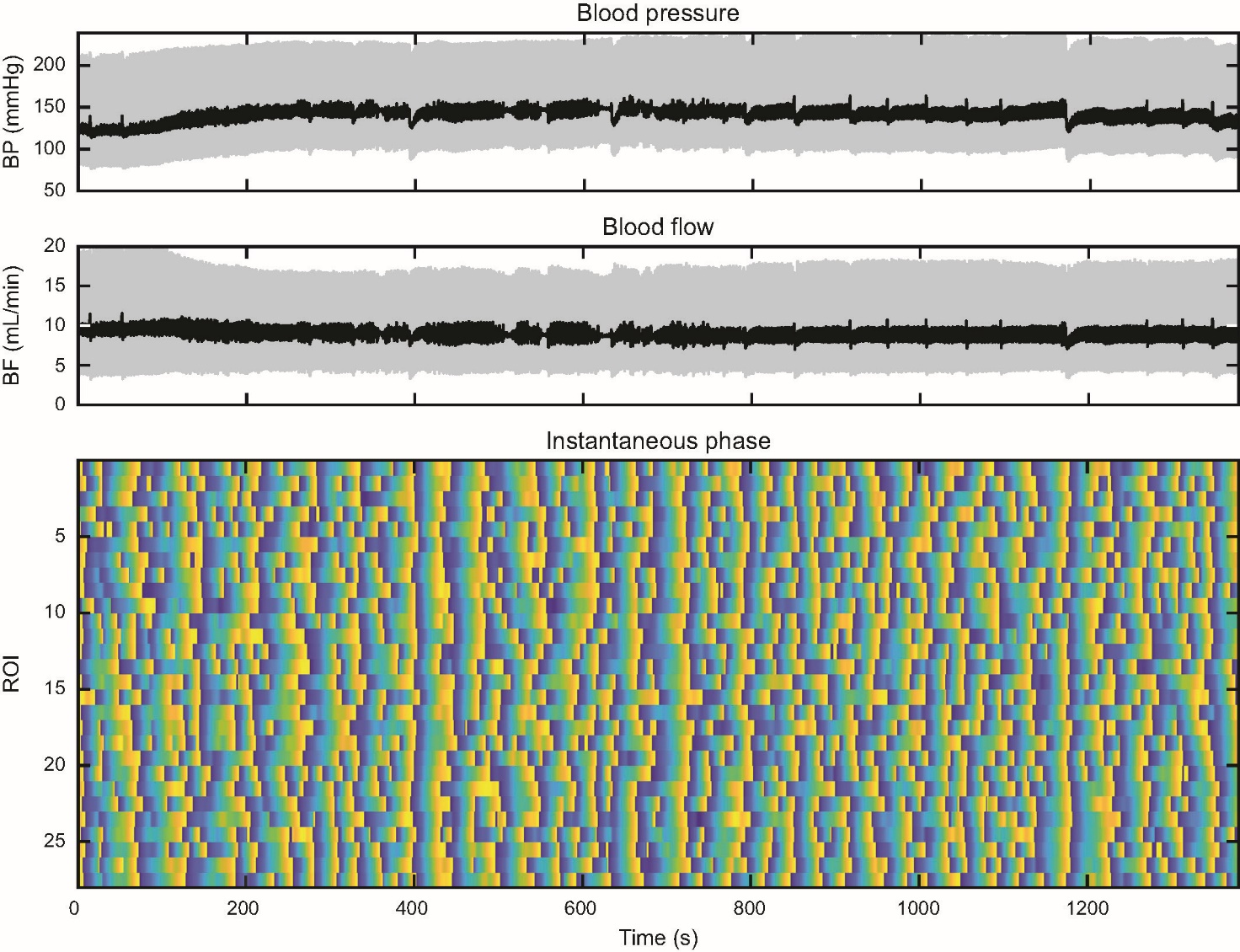


SUPPLEMENTAL **Figure 2**.**A**) This WT rat shows evidence of synchronization in the first 400 s based on partial entrainment, on similarity of TGF cycle duration, and on the lack of response of RBF to the small BP transients. It then decays and restarts at 500 s in ROIs 18 – 30 before spreading into all ROIs at ≈750 s and continuing until the end of the record.


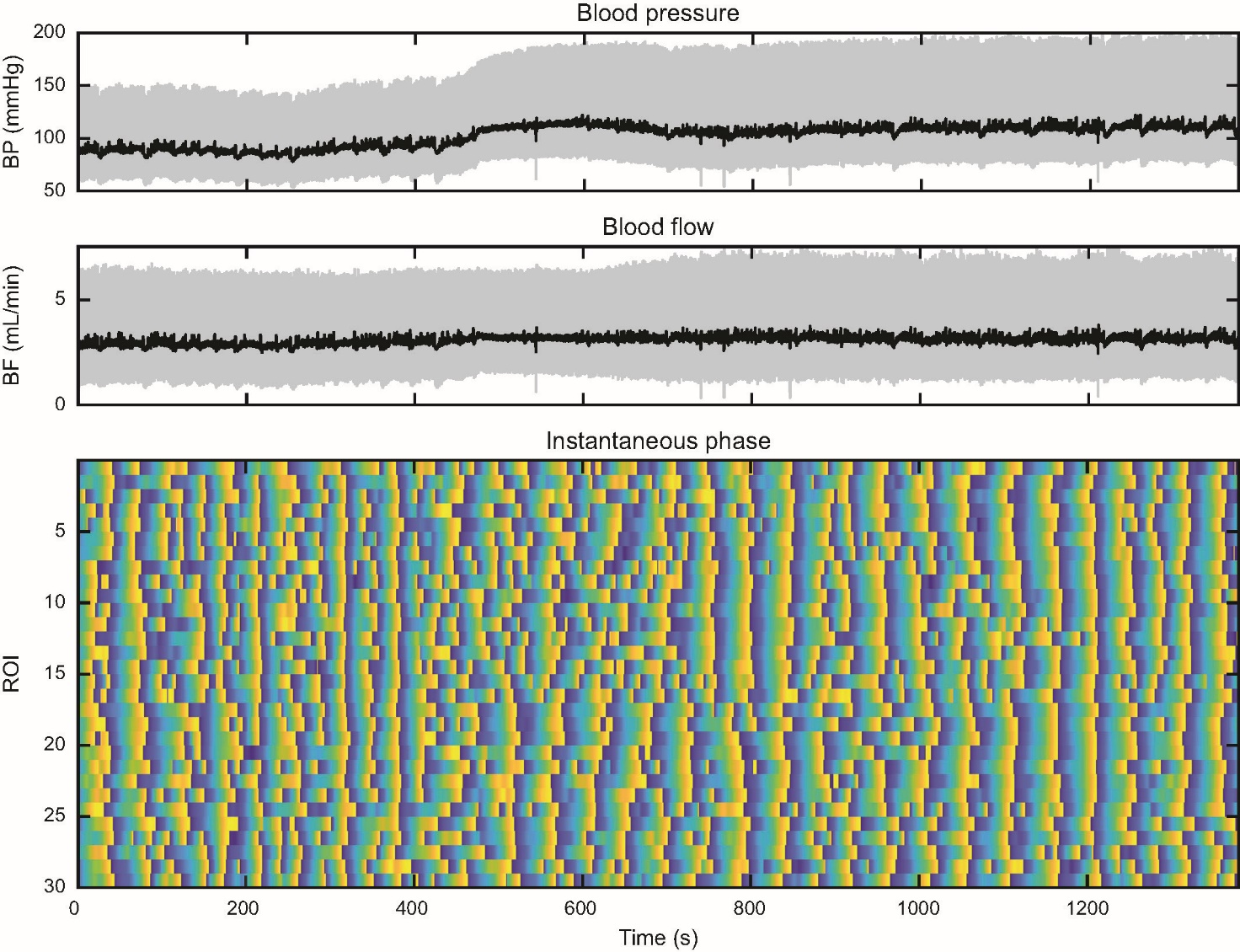


SUPPLEMENTAL **Figure 2.B**) This KO rat shows only one entrained TGF cycle, at 480 s, in the entire record. In the absence of BP fluctuation, there is remarkably little TGF entrainment in this record.


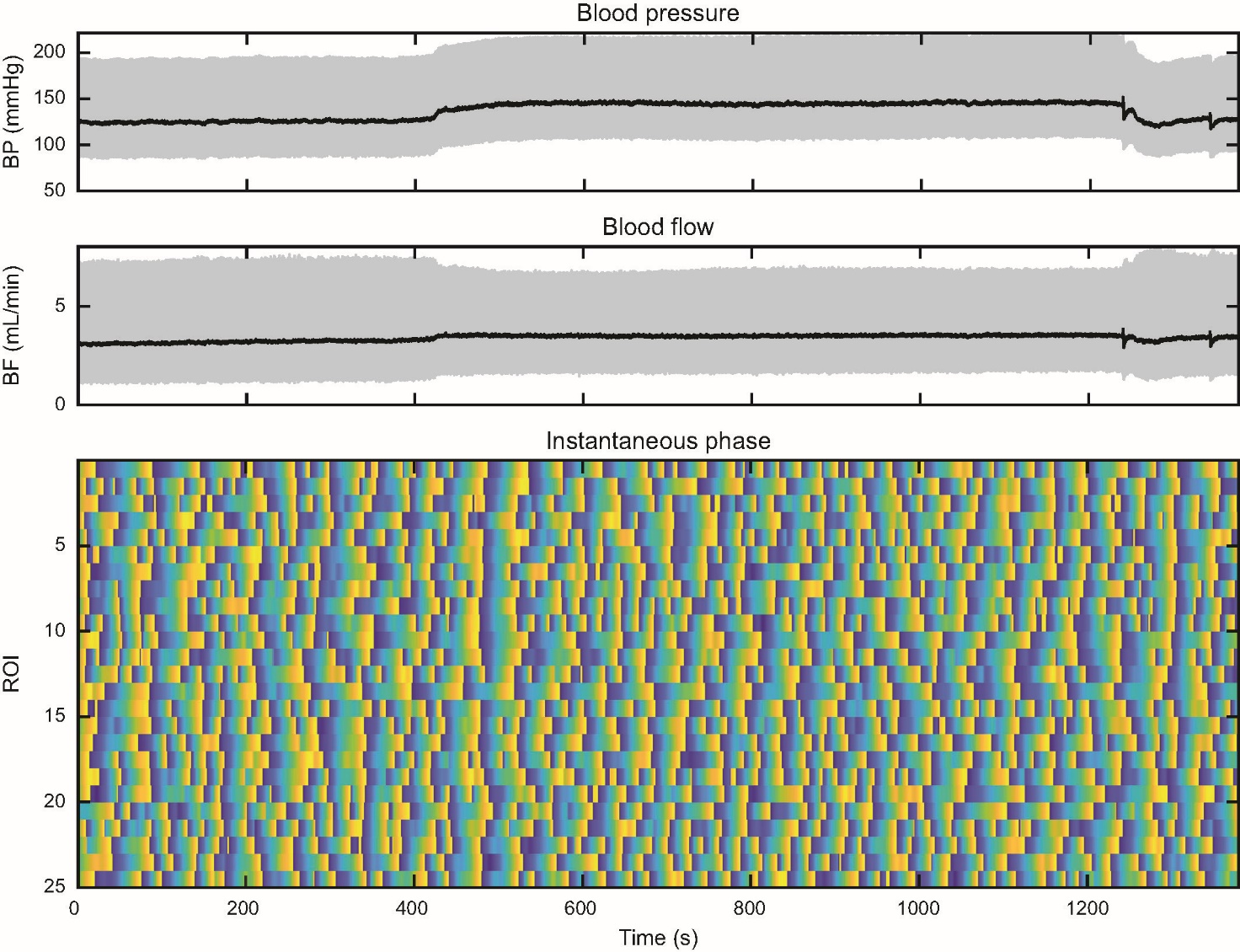


SUPPLEMENTAL **Figure 3.A**) This WT rat shows numerous, substantial BP transients from the start of the record to 650 s with smaller, fewer BP excursions thereafter. Extensive TGF synchronization involving almost all ROIs (1 – 24) is evident throughout the record. This occurs despite considerable transient fluctuation of BP. Even the stronger BP events at ≈50 s, ≈470 s, and at ≈575 s do not perturb TGF synchronization.


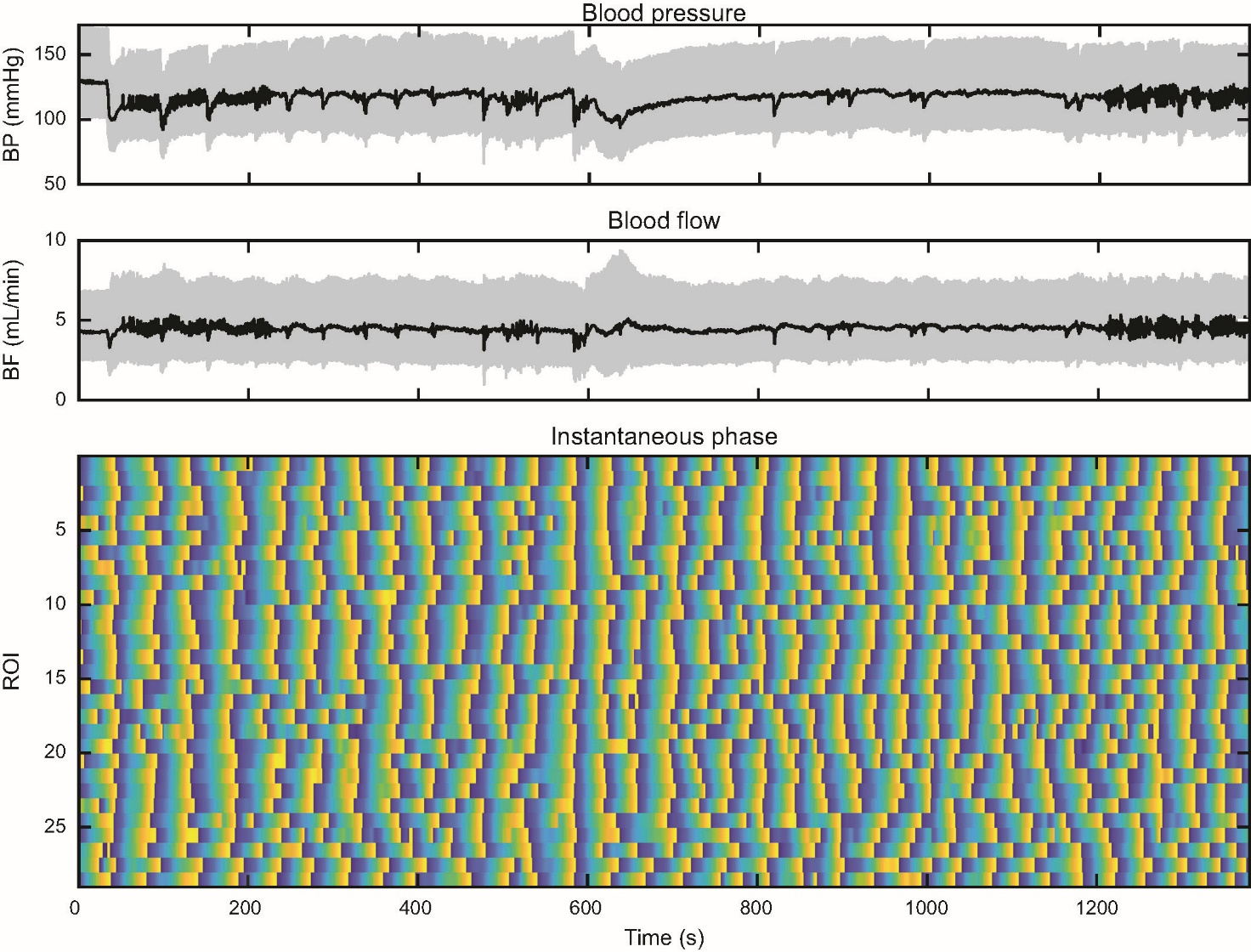


SUPPLEMENTAL **Figure 3.B**) This KO rat shows multiple BP events that are similar in magnitude to those seen in the WT rat shown in (Figure **3.A**). In the KO rat these provoked strong entrainment of TGF. Only before the first BP transient at 170 s and after 890 s when the BP transients decrease in amplitude is the TGF entrainment reduced. Note also that there is considerable variation in the apparent TGF cycle duration.


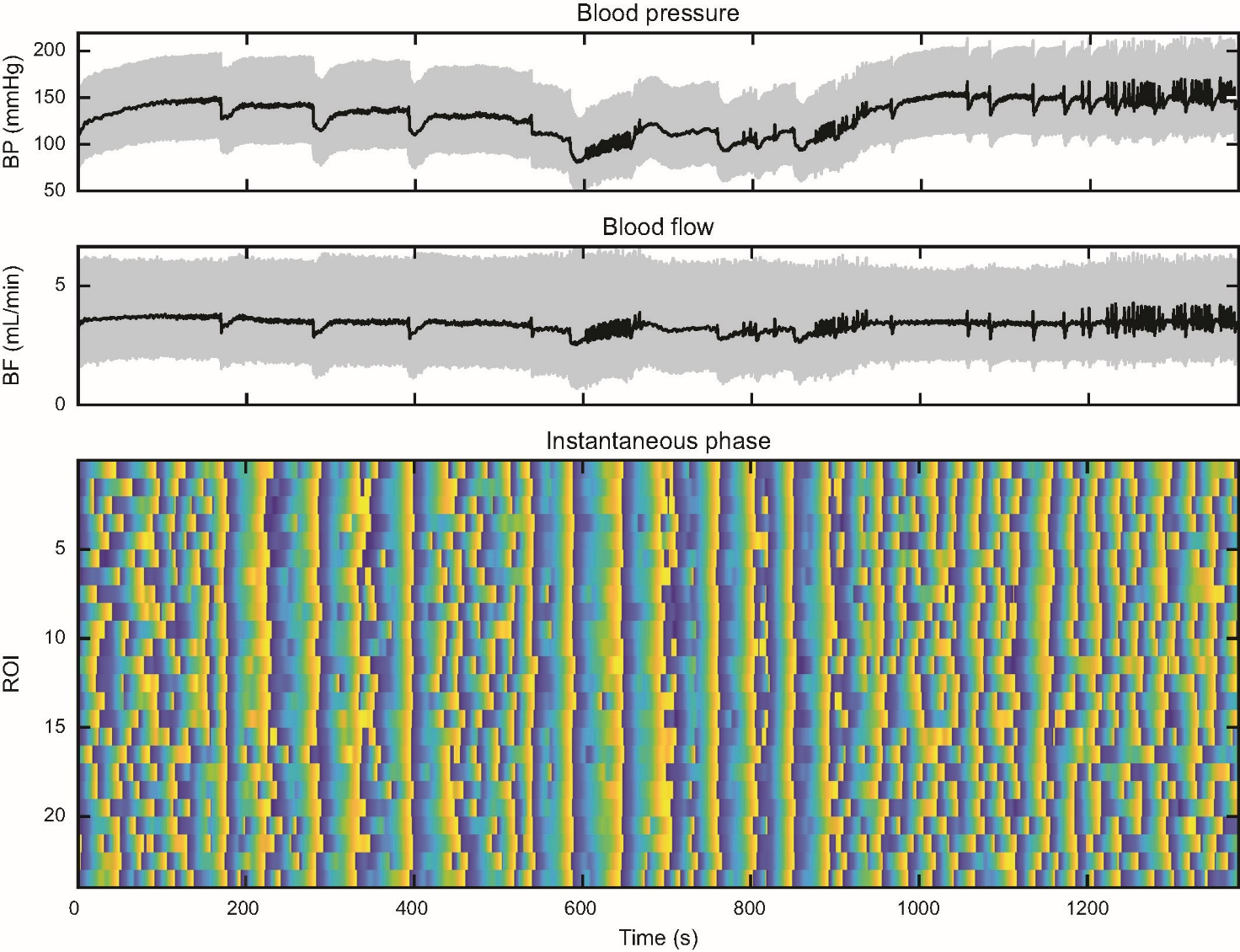

Supplement: Supplementary file 1 [file Table1.DOCX]
